# Boosting the Tunable Microwave Scattering Signature of Sensing Array Platforms Consisting of Amorphous Ferromagnetic Fe_2.25_Co_72.75_Si_10_B_15_ Microwires and Its Amplification by Intercalating Cu Microwires

**DOI:** 10.3390/nano11040920

**Published:** 2021-04-04

**Authors:** Diego Archilla, Jesús López-Sánchez, Antonio Hernando, Elena Navarro, Pilar Marín

**Affiliations:** 1 Instituto de Magnetismo Aplicado, UCM-ADIF, 28230 Las Rozas, Spain; diegoarc@ucm.es (D.A.); antherna@fis.ucm.es (A.H.); enavarro@ucm.es (E.N.); mpmarin@ucm.es (P.M.); 2 Spanish CRG BM25-SpLine at The ESRF—The European Synchrotron, 38000 Grenoble, France; 3 Instituto de Ciencia de Materiales de Madrid (ICMM), CSIC, 28049 Madrid, Spain; 4 Institutos Madrileños de Estudios Avanzados (IMDEA) Nanociencia, 28049 Madrid, Spain; 5 Donostia International Physics Centre (DIPC), 20018 Donostia, Spain; 6 Escuela Politécnica Superior, Universidad Antonio de Nebrija, Calle de los Pirineos, 55, 28040 Madrid, Spain; 7 Departamento de Física de Materiales, Universidad Complutense de Madrid (UCM), 28040 Madrid, Spain

**Keywords:** soft magnetic materials, amorphous magnetic materials, magnetic microwires, microwave scattering, sensing array platforms

## Abstract

The following work addresses new configurations of sensing array platforms that are composed of Co-based amorphous ferromagnetic microwires (MWs) to obtain an enhanced modulation of the microwave scattering effects through the application of low strength DC or AC magnetic fields. An amorphous MW is an ultrasoft ferromagnetic material (coercivity ~0.2 Oe) with a circumferential magnetic anisotropy that provides a high surface sensitivity when it is subjected to an external magnetic field. Firstly, microwave scattering experiments are performed as a function of the length and number of MWs placed parallel to each other forming an array. Subsequently, three array configurations are designed, achieving high *S*_21_ scattering coefficients up to about −50 dB. The influence of DC and AC magnetic fields on *S*_21_ has been analyzed in frequency and time domains representation, respectively. In addition, the MWs sensing array has been overlapped by polymeric surfaces and the variations of their micrometric thicknesses also cause strong changes in the *S*_21_ amplitude with displacements in the frequency that are associated to the maximum scattering behavior. Finally, a new concept for amplifying microwave scattering is provided by intercalating Cu MWs into the linear Co-based arrays. The designed mixed system that is composed by Co-based and Cu MWs exhibits a higher *S*_21_ coefficient when compared to a single Co-based MW system because of higher electrical conductivity of Cu. However, the ability to modulate the resulting electromagnetic scattering is conferred by the giant magneto-impedance (GMI) effects coming from properties of the ultrasoft amorphous MWs. The mixed array platform covers a wide range of sensor applications, demonstrating the feasibility of tuning the *S*_21_ amplitude over a wide scattering range by applying AC or DC magnetic fields and tuning the resonant frequency position according to the polymeric slab thickness.

## 1. Introduction

Exploring new physicochemical phenomena that arise, when materials are confined to the nanoscale, as surface energy becomes relevant or dominates the volume energy, is one of the fundamental strengths of the multifunctional applied materials science. However, there are different compositional and shape configurations that belong to the microscale that dazzle by their high versatility and variability in their properties, when they are subjected to an external stimulus (i.e., an applied electric and/or magnetic field, mechanical stresses, temperature, and a gas exposure) [1,2,3,4,5,6,7]. Specifically for the electromagnetic microwave technology, multifunctional materials with tunable properties by external stimuli are essential to progression in the field of sensing applications [4,8,9], microwave shielding systems [7,10,11,12], wireless communication [13,14], antenna engineering [15,16], hyperthermia [17], and biomedical engineering [18,19,20]. The crossed control of the electromagnetic properties is appealing for advanced performances, involving the ability of modulating the electrical response by means of a magnetic field and conversely. Such cross-modulation requires materials with strong magneto-electric coupling [21].

In this scenario, Fe_2.25_Co_72.75_Si_10_B_15_ amorphous ferromagnetic microwires (Co-based MWs) are excellent candidates for hosting the multifunctionality for microwave sensing applications, because they exhibit a high-frequency giant magneto-impedance (GMI) effect, being highly sensitive to those external stimuli [2,3,22]. The Co-based microwires (MWs) with a small addition of Fe or Mn present a near-zero negative magnetostriction of the order of 10^−7^ and show a predominant circumferential magnetic anisotropy [23]. The latter is the result of the coupling between the negative magnetostriction and the large internal tensile along the MWs induced by the Taylor–Ulitovsky technique [24,25,26,27]. N.A. Usov et al. [28] theoretically demonstrated for the first time a giant GMI effect with a peak in the longitudinal component of the MW impedance at the magnetic anisotropy field (*H*_K_), when the easy direction of the magnetization is circumferential. Since then, many works have been made studying the evolution of their mechanical, electrical, and magnetic properties as a function of a thermal annealing [29], mechanical stress under annealing [30,31], and twisting the MW a certain angle [32] (among others). Furthermore, tunable and enhanced physical properties are obtained when the MWs are distributed in an array with interesting and powerful results [29,33,34,35]. In this line, wireless systems that are composed by MWs with GMI effects in the microwave range have been recently reported [36]. In the presence of electromagnetic waves, the frequency that is associated to the maximum scattering is determined by the length of the MW through a dipole electric resonance [37]. The scattering coefficient (*S*_21_) can be modulated by means of the application of a DC or AC magnetic field in the presence of GHz-ranged electromagnetic wave due to the GMI effect observed in Co-based MWs.

The present work is conceived to identify novel configurations of MW arrangement to obtain strong microwave scattering effects adjustable by DC or AC magnetic fields. In such a scenario, a parallel Co-based MWs arrangement (equidistantly spaced) presents substantial improvements of the scattering coefficient *S*_21_ as a function of their distribution and number of MWs. The physical mechanism behind is based on the high-frequency electric current that is induced in the MW due to the incident electric field component (***E***^inc^) and parallel to the axis of the MW coming from the electromagnetic wave with frequencies in the GHz range. Here, the MW polarization depends strongly on the surface impedance and the magnetic skin depth (*δ*_m_) near the MW resonance. In addition, it can be modulated, varying their magnetic domain structure by an external low strength magnetic field [38,39]. In turn, advanced sensing devices must generate a large scattering response with a resonant sharp behavior to monitor small variations in the scattering spectra accurately. To do this, the electromagnetic scattering measurements are also performed in time domain representation to avoid parasitic electrical error contributions in order to obtain a correct interpretation of the results [33].

In other works, experiments involving microwave scattering have been carried out in composites with Cu-based MW fillers, a metallic material. Inside them, interesting values of *S*_21_ (~−20 dB) with volume fractions that are lower than 1% are obtained [40]. Therefore, their ability to improve the powerful responses obtained for the Co-based parallel arrays is also investigated. As a novel strategy, Cu MWs are intercalated between the Co-based ones with the same length (*L* = 4 cm). The resulting scattering features are enhanced with respect to the single Co-based system. In addition, a modulation of the signal of the designed system is achieved when applying the AC or DC magnetic fields. This combination of equidistantly spaced MWs opens up the possibility of tuning and boosting the microwave scattering properties of the existing systems due to the GMI effects of the Co-based MWs and the higher electrical conductivity of Cu MWs. The results that are presented in this work are promising for the fabrication of artificial microstructures, named metamaterials, which are used in the control of the electromagnetic waves [41]. The traditional approach to building metamaterials requires the combination of two different arrays to produce a double negative response (electric permittivity and magnetic permeability [42]) in the same frequency range [41]. However, the use of single arrays that are composed by ultrasoft magnetic MWs enables a bias field control of the response [43,44,45,46] into the Cu MWs, presenting a new configuration that follows the same line as the split ring resonators or other types of currents loops in the design of double negative medium [41,43].

## 2. Materials and Methods

### 2.1. Fabrication and Characterization of the Co-Based Amorphous Ferromagnetic MWs

Amorphous ferromagnetic Fe_2.25_Co_72.75_Si_10_B_15_ MWs are fabricated with a Pyrex (borosilicate glass) shell by the modified rapid-quenching Taylor–Ulitovsky technique [24,25,26,27]. For the present work, a metallic core diameter of 31 µm and a total diameter of 50 µm are employed. Considering these characteristics, the Co-based MWs exhibit a low negative magnetostriction with a dominant circumferential magnetic anisotropy, which results in an ultrasoft hysteresis loop (*H*_C_ ~ 0.2 Oe) with high permeability [47]. Therefore, a high-sensitivity GMI effect is expected, when an axial magnetic field is applied, coming from large changes in the surface impedance [48].

Morphological features and structural properties are examined by scanning electron microscopy (SEM, JEOL Ltd, Tokyo, Japan) while using a JEOL JSM 6400 system. The crystal structure is studied by X-ray diffraction (XRD, Malvern Panalytical, Almelo (Netherlands) and Malvern (United Kingdom)) in a Bragg–Brentano configuration using a PANalytical X’Pert MPD apparatus with Cu Kα radiation (*λ* = 1.542 Å). The magnetic properties of the MWs are carried out using a Physical Property Measurement System Vibrating Sample Magnetometer (PPMS-VSM model 6000 controller, Quantum Design International, San Diego, United States) to obtain hysteresis loops under a maximum applied magnetic field of 1 T at room temperature (25 °C).

### 2.2. Design of the Sensing Platforms Composed of Co-Based and Co-Based + Cu MWs Linear Arrays

Three configurations are fabricated to examine their microwave scattering properties.

- **Configuration 1:** one sensing platform composed by a 15 MWs linear array. The MWs have a length of 4 cm and they are equidistantly separated by 0.5 cm.
- **Configuration 2:** one sensing platform composed by three columns of 15 MWs linear array. The MWs have a length of 4 cm, and they are separated equidistantly by 0.5 cm. The spacing between columns is also set to 0.5 cm.
- **Configuration 3:** two sensing platforms of the configuration 2 in a tandem form.

### 2.3. Operational Physical Basis and High-Frequency Characterization

Metallic MWs interact with the electromagnetic waves as micro-antennas with a resonance that is defined by the effective permittivity of the surrounding medium (*ε*_eff_) and by the length (*L*) of the order of cm in the GHz range [2]. However, in the case of Co-based MWs, the possibility of magnetically tuning the present dipole resonances is conferred by the high-permeability of the MWs when their radius (*r*) are in the same order of the magnetic skin depth (*δ*_m_ ~ µm). Therefore, the *r ~ δ*_m_ condition ensures a good interaction between the electromagnetic waves and ferromagnetic core.

The arrays are built by hand with an equidistant distance between the MWs of 0.5 cm, and they are placed in a plastic plate (dielectric substrate) with a thickness (*d*) of 700 µm to ensure the structural stability of the arrays. The fixed distance between MWs is chosen according to the reported theoretical study in which an enhanced GMI effect is obtained with MWs with a diameter of 30 µm [33]. The MWs were clamped in the plastic sheet without stress by a commercial tape to avoid changes in the permittivity media during the microwave experiments.

The microwave scattering properties of the MWs were experimentally measured using two double-ridged guide horn antennas with an operation frequency from 800 MHz to 18 GHz. The distance between the horn antennas is 1.2 m, with the sample being placed at 0.6 m from the emitting and the receiver antenna (equidistant point). Because the transmission electromagnetic horn antennas are in the far-field range, the electromagnetic waves can be considered as plane waves with a propagation constant **k** towards the MWs array. The antennas were connected to a programmable network analyzer (Agilent E8362B PNA Series Network Analyzer), and the transmission coefficient *S*_21_ that gives the relation between the power of the emitting antenna (*P*_1_) and the power of the receiving antenna (*P*_2_) is measured after calibration procedures that are based on open-air measurements (Equation (1)). This coefficient is directly related with the electrical current induced along the MW axis by the incident wave, since the scattered wave is generated by this current [36,46].

(1) $$S_{21}=20{\;\log}_{10}\frac{P_{2}}{P_{1}}$$

The magnetic field of the incident microwave radiation is oriented perpendicular to the MW axis, whereas the electric field is parallel to this axis to obtain both a polarization and a current induction along the MW axis. In turn, two Helmholtz coils (current, *I* = 0.2 A and operating frequency *f*_operating_ = 10 Hz) are used to apply both DC and AC magnetic fields parallel to the axis of the MWs to study the influence of their magnetic response on the microwave scattering spectra in the frequency and time domain representations. The distance between the two Helmholtz coils is 0.4 m, the same length as the radius of the coils. Those that are applied DC or AC magnetic fields are the controlling factors of the microwave spectrum, since the surface impedance is related with the MW permeability that depends on its magnetic structure [46,49].

**Figure 1:**
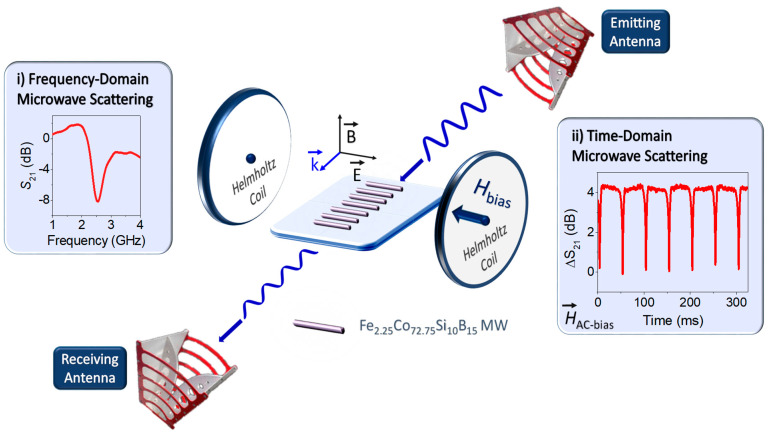
Schematic representation of the microwave scattering mechanism in transmission mode for sensing array platforms composed by Fe_2.25_Co_72.75_Si_10_B_15_ (lengths of a few cm). Two Helmholtz coils apply axial magnetic fields to induce large changes in the magnetic state of the Co-based microwires (MWs) and then, giant magneto-impedance (GMI) effects arises. The *S*_21_ transmission coefficient is obtained in the (**i**) frequency and (**ii**) time domains.

To conclude the experimental section, two types of measurements are performed in the microwave scattering experiments: in the frequency and time domains. Figure 1 shows an illustration of the microwave scattering experimental setup in transmission mode. In the frequency domain, the *S*_21_ scattering coefficient shows a minimum at the dipole resonance frequency (*f*_min_) [35], which is related to the electric permittivity of the surrounding media of the MWs *(ε*_d_), MW length (*L*), and speed of light (*c*) by the Equation (2). *S*_21_ coefficient was modulated by applied DC-magnetic fields from 0 to 17 Oe, where these values are previously calibrated with a Hall probe.

(2) $$f_{\min}=\frac{c}{2L\sqrt{\varepsilon_{d}}}$$

In the time domain representation, the ∆*S*_21_(t) evaluation is performed at a fixed frequency localized in the close spectral environment of the dipole resonance. This measurement is carried out with an applied axial AC-magnetic field of 17 Oe at 10 Hz produced by the Helmholtz coils. The ∆*S*_21_ pulses are generated continuously, coming from the changes of the surface impedance of the MW when the magnetization and demagnetization processes are induced by the mentioned applied AC-field. In addition, the influence of the thickness of overlapping polymeric slabs (*t*) on the sensing array platforms is investigated to experimentally evaluate the matching thickness of the optimized microwave scattering systems.

## 3. Results and Discussion

### 3.1. Structural and Magnetic Characterization of Co-Based MWs

**Figure 2:**
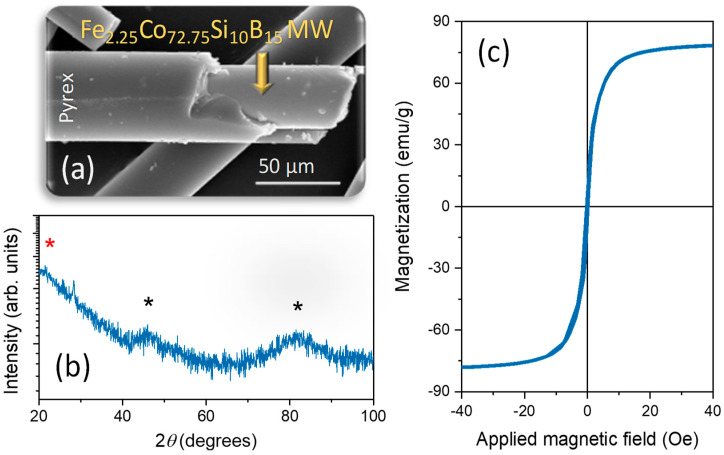
(**a**) The scanning electron microscopy (SEM) image, (**b**) X-ray diffraction (XRD) pattern, and (**c**) magnetic hysteresis loop of Co-based MWs. The amorphous contributions of the Pyrex and the Co-based alloy are indicated by red and black asterisks, respectively, in Figure 2b. The hysteresis loop is performed in one single Co-based MW at 25 °C.

Figure 2a shows a SEM image, where the MW microstructure that is composed of a metallic core and the outermost Pyrex layer is clearly identified. In turn, the XRD pattern (Figure 2b) displays broad amorphous shoulders located one below 30°, and two around ~45° and ~82°. The first contribution corresponds to the Pyrex XRD signature (red asterisk) [37] and the other contributions (black asterisks) are associated with the amorphous Co-based alloys [50]. Regarding the magnetic properties, a high saturation magnetization of ~81 emu/g (that is normalized to the magnetic mass) and an extremely low coercivity around ~0.2 Oe (Figure 2c) are shown. Before saturation condition, the magnetization exhibits a linear dependence on the axially applied magnetic field, as can be expected from its circumferential anisotropy. In this context, the present amorphous characteristics confers a magnetic domain structure that is determined by the magnetoelastic anisotropy arising from the coupling of the negative magnetostriction characteristic of the Co(Fe)-based composition and the mechanical stresses that formed during the manufacturing process maintained by the external cover of Pyrex [50].

### 3.2. Evolution of the Microwave Scattering Properties in Co-Based MWs Linear Arrays

**Figure 3:**
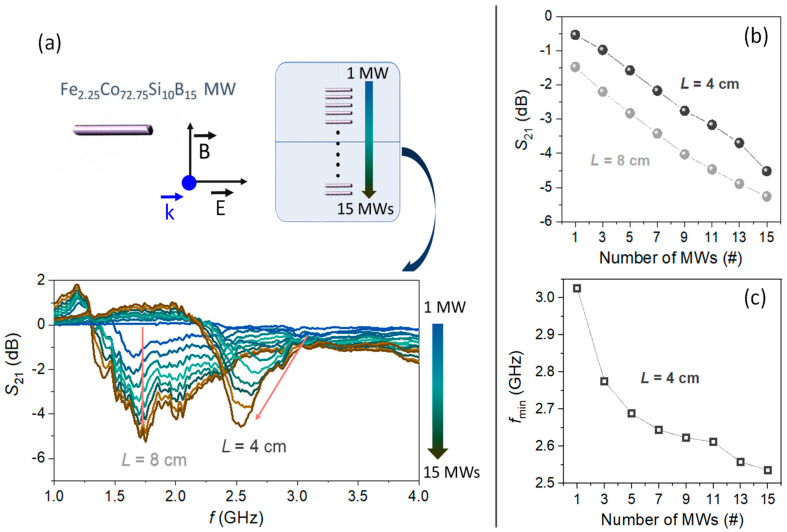
(**a**) Top part illustrates the sensing array platform layout from 1 to 15 Co-based MWs. Bottom part shows the *S*_21_ transmission coefficients in the L and S microwave spectral GHz regions obtained for two linear arrays composed of Co-based MWs with a length of 4 cm and 8 cm from 1 to 15 MWs. (**b**) *S*_21_ minimum value and (**c**) *f*_min_ as a function of the number of 4 cm MWs in the array collected from the results showed in the bottom of (**a**).

In previous works, the position of the dipole resonance varies with the length *L* of the MW, while considering Equation (2) [48,51]. In accordance with those, lengths of 4 cm and 8 cm are chosen to evaluate the microwave scattering behavior of the MWs, when they are arranged in a linear array. Figure 3a shows how the *f*_min_ that is associated to the maximum scattering for a single MW falls around 3.03 GHz and 1.64 GHz for the lengths of 4 cm and 8 cm, respectively. Accordingly, their maximum scatterings are located in the S-band (short wave region) and in the L-band (long wave region). As the number of parallel MWs increases, noticeable changes are detected in the intensity, bandwidth, and position of the minimum *S*_21_. Specifically, the |*S*_21_| coefficient at *f*_min_ of both array configurations shows an almost linear trend with an increase of −3.8 dB and −4.0 dB for 4 cm and 8 cm length, respectively, from 1 MW to 15 MWs (Figure 3b).

These experiments confirm a behavior equivalent to that observed in antenna arrays widely used in point-to-point communication systems, where a very high directive beam of radiation is needed [52]. In our previous work [48], it demonstrates how, with parallel MWs equidistantly placed at a constant distance of *λ*/2, the radiation is concentrated in one axis becoming a more directional scattering of the electromagnetic wave coming from the emitting antenna as the number of MWs increases from 1 to 4. The MWs behave as a dipole antenna with a resonance at *λ*/2 condition resulting in an enhancement of the S_21_ scattering parameter [48]. Therefore, these results reveal no MWs length dependence for the gain obtained. Instead, while the *f*_min_ of 8-cm-length MWs barely varies with the number de MWs, *f*_min_ shifts from 3.03 GHz to 2.54 GHz for 4-cm-length MWs (Figure 3c). The present results prove that these simple system configurations are potentially interesting for tuning the frequency of the bandpass.

### 3.3. Microwave Scattering Experiments Performed in Co-Based MWs Sensing Array Platforms by Applying DC/AC Magnetic Fields

The possibility to tune the scattering parameter *S*_21_ with an external DC magnetic field is an important property of these array configurations [2]. The surface impedance tensor, $\hat{\varsigma }$, of MWs is linked to the tangential components of the electric, $E_{t}$, and the magnetic, $H_{t}$, fields at the MW surface [2]:

(3) $$E_{t}=\hat{\varsigma }\;\left(H_{t}\times n_{r}\right)$$

with $n_{r}$ the unit radial vector directed through the MW. The diagonal component of the surface impedance tensor, $\varsigma_{zz}$, relates the longitudinal electric field $E_{Z}$ and circular magnetic field $H_{\varphi }$ created by the induced current in the MW [53]:

(4) $$E_{Z}=\varsigma_{zz}\;H_{\varphi }$$

In the case of the MW radius $r\sim \delta_{m}$, the electric polarization can be modulated by the MW magnetization and $\varsigma_{zz}$ has the form [53]:

(5) $$\varsigma_{zz}=\;\frac{c\left(1-j\right)}{4\pi \sigma \delta }\left(\sqrt{\tilde{\mu }}\;\cos^{2}\theta +\;\sin^{2}\theta \right)$$

with *θ* the angle between the uniform magnetization vector and the MW axis, σ the electrical conductivity of the MW, $\delta =c/\sqrt{2\pi \sigma \omega }$ the electric skin depth, $\tilde{\mu }$ the dynamic permeability ($\tilde{\mu }=\frac{\;\partial M}{\partial H}$), and $\delta_{m}=c/\sqrt{2\pi \sigma \omega \tilde{\mu }}$ the magnetic skin depth. The surface impedance depends on both the dynamic permeability and the magnetization orientation angle θ, according to Equation (5). Therefore, the electric polarization and current distribution depend on the surface impedance and, hence, on the magnetization direction through $\tilde{\mu }$ [53]. Applying an axial DC magnetic field, the magnetization changes from circumferential to longitudinal orientation (*θ* in Equation (5) changes from π/2 to 0), and that produces a strong variation on the surface impedance of the Co-based MW at the GHz frequencies. The increase in permeability occurs in the magnetization reorientation, which leads to an increase of the surface impedance and, then, it results in a reduction in the induced current in the MW. The reduction in the power absorbed by the MW produces a smoothing in the *S*_21_ parameter [43,51].

**Figure 4:**
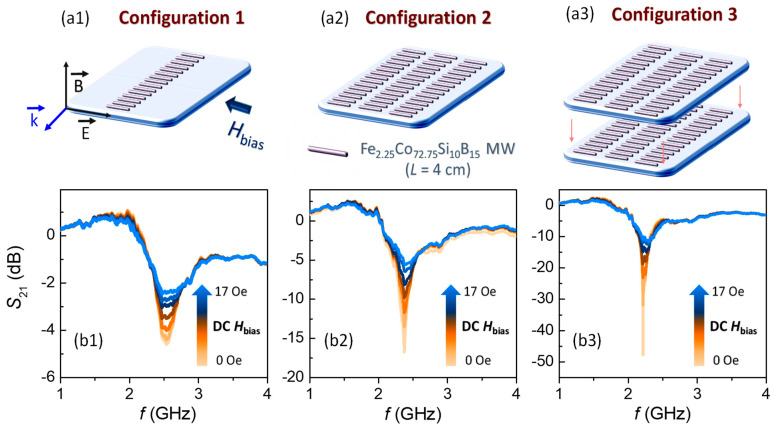
Effect of an external DC magnetic field with increasing value up to 17 Oe in the microwave scattering spectrum: (**a1**–**a3**) schematic illustration of MWs configurations and (**b1**–**b3**) *S*_21_ transmission coefficient obtained for the configurations 1, 2, and 3.

Linear array configurations 1, 2, and 3 are employed to investigate the impact of the above phenomena on the microwave scattering spectrum (Figure 4a1–a3, respectively).

Subsequently, microwave scattering experiments are performed as a function of a DC magnetic field varying from 0 to 17 Oe (Figure 4b1–b3). In the condition of *H*_bias_ = 0 Oe, the configuration 1 (Figure 4a1) displays a minimum intensity of the *S*_21_ coefficient of ~−4.6 dB that is located around *f* ~ 2.54 GHz (full width high maximum, FWHM = 0.30 GHz). When the system is extended to three linear arrays (Figure 4a2), the minimum intensity continues to increase up to ~−16.8 dB. In addition, a shift in the frequency of the minimum scattering is noticed reaching ~2.37 GHz and its bandwidth is becoming much narrower (FWHM = 0.17 GHz). Interestingly, the most notable changes are observed when two sensing platforms are arranged in tandem (Figure 4a3), since a huge enhancement of the *S*_21_ minimum intensity up to ~−47.9 dB is recorded (Figure 4b3). These values are highly competitive [37], since an enormous increase of the *S*_21_ coefficient of ~1041% is achieved by changing from configuration 1 to 3. In turn, a drastic narrowing of the scattering is also registered (FWHM = 0.03 GHz), shifting the minimum frequency to ~2.21 GHz. Therefore, configuration 3 also enables accurately tracking shifts in the transmission spectra, one of the required criteria for a sensing device.

**Figure 5:**
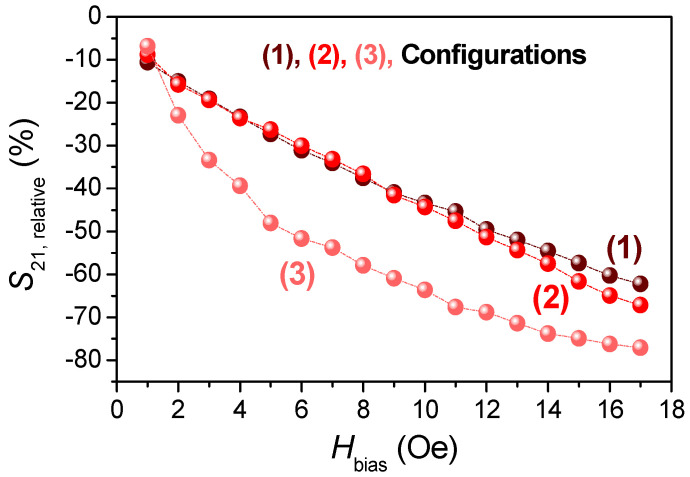
Representation of the *S*_21, relative_ as a function of the DC axial magnetic field from 1 to 17 Oe for the three linear array configurations represented in Figure 4a1–a3.

As the magnetic DC field is increased from O to 17 Oe, the *S*_21_ coefficient is strongly flattened for all of the analyzed configurations. The most remarkable change is found in configuration 3, with a progressive increase from ~−47.9 to ~−11.6 dB (Figure 4b3). The applied DC magnetic field increases the permeability of the MWs, which, in turn, is closely related to their impedance. In Figure 4b1–b3, these effects are noted gradually up to a maximum applied magnetic field of 17 Oe (a value near the anisotropic field according to Figure 2c). Moreover, DC magnetic fields also affect the bandwidth, which increases by one order of magnitude when the field is present (for example, configuration 3 goes from ~0.03 GHz at *H*_bias_ = 0 Oe to ~0.34 GHz at *H*_bias_ = 17 Oe). The field-based broadening is also observed in composites that contain short Co-based MWs [29]. In contrast, the *f*_min_ is barely modified by this effect due to its electric dipole character (Equation (2)). To compare the evolution of the field-dependence of the *S*_21_ coefficient obtained from the three configurations, its relative variation (*S*_21,relative_) is represented as a function of the DC applied magnetic field shown in Figure 5.

*S*_21,relative_ is defined, as follows:

(6) $$S_{21,\mathrm{relative}}\left(\%\right)=100\;\times \;\frac{{\left|S_{21}\right|}_{H\neq 0\;\mathrm{Oe}}\;-\;{\left|S_{21}\right|}_{H=0\;\mathrm{Oe}}}{{\left|S_{21}\right|}_{H=0\;\mathrm{Oe}}}$$

For the configurations 1 and 2, a decreasing linear trend of the *S*_21,relative_ is found with the applied DC magnetic field. Increasing the number of Co-based MWs from 15 to 45 arranged in three linear array columns results in a moderate enhancement of the GMI effect with a maximum gain from ~62% for configuration 1 to ~67 % for configuration 2. In contrast, configuration 3 exhibits a decreasing logarithmic trend, reaching a large relative variation of ~77%. Therefore, the GMI effect is more noticeable in the tandem system. The presented results show a high performance, obtaining a magnetic field-tunable microwave spectrum, but, also, it might be used as sensitive wireless sensors to remotely measure low strength magnetic fields.

**Figure 6:**
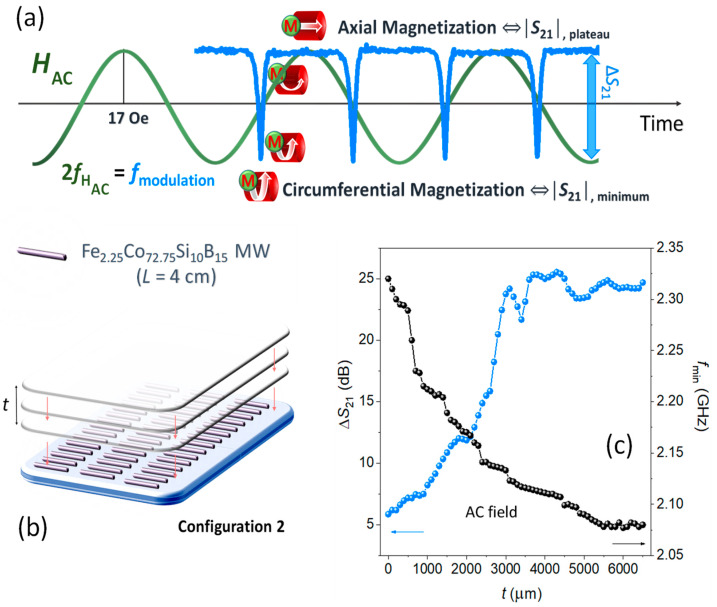
AC magnetic field studies performed in the sensor array platform configuration 2: (**a**) Illustration of the influence of the magnetization switch from MWs with a circumferential magnetization (*S*_21,minimum_) to one with an axial magnetization (*S*_21,plateau_) produced by a saturating AC magnetic field (*H*_AC_). The frequency of the modulation (*f*_modulation_) is twice the frequency of *H*_AC_ (*f*_HAC_). (**b**) Layout of the configuration 2 with overlapping polymer slabs (*t*). (**c**) The ∆*S*_21_ and *f*_min_ parameters as a function of the total thickness of polymer slabs (*t*).

An advanced step of the variations in the microwave scattering spectra consists in applying an AC magnetic field to induce periodic changes in the internal structure of the MWs magnetic domains, in this case, modulated in time. The following study can be performed for any of the three configurations. As an example of application, a 17-Oe-amplitude magnetic field with a frequency of 10 Hz (near the saturation field) is applied to configuration 2. This relatively low strength magnetic field that is produced by the Helmholtz coils enables the magnetization to oscillate between a circumferential and axial direction periodically (Figure 6a).

Specifically, the maxima and minima of the AC magnetic field wave cause the magnetization to be saturated along the MW axis (upper plateau of the *S*_21_ coefficient). Instead, the zero values of this AC wave cause remanence state conditions (circumferential anisotropy) that correspond to the minima of *S*_21_ values. The GMI phenomena are notorious between two states, where the dynamic permeability change and that correspond to both the sharp negative and positive slopes of the modulated *S*_21_ signal around the minima. In addition, for a single wavelength of the displayed signature, its preferential anisotropy state is changed twice, because the hysteresis loop is symmetrical (Figure 2c). According to this, the modulation frequency (*f*_modulation_) of *S*_21_ parameter is twice the frequency of the applied magnetic field (*f*_HAC_). The amplitude of the modulation (pulse depth) is given by the difference of the two magnetic states (∆*S*_21_ = |*S*_21,minimum_| − |*S*_21,plateau_|). The influence of ∆*S*_21_ modulation is subsequently studied as a function of the thickness of polymeric slabs (*ε*_r_~3) up to 6000 µm (*t*) overlapping on top of array platform configuration 2 (Figure 6b).

**Figure 7:**
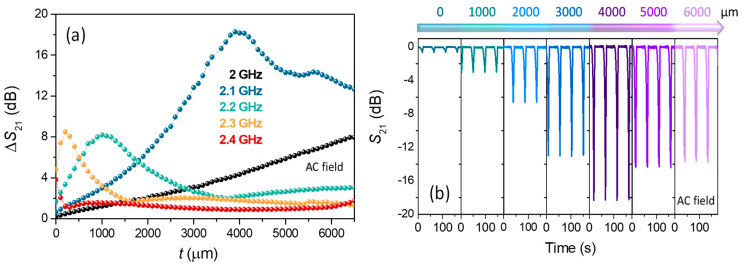
AC magnetic field experiments performed in the sensor array platform configuration 2 of Co-based MWs: (**a**) the ∆*S*_21_ trends with the overlapped thickness placed on the top of the Co-based MWs at fixed frequencies ranged from 2.00 GHz to 2.40 GHz; (**b**) time-resolved modulation of the scattering coefficient ∆*S*_21_ due to an AC-bias magnetic field (*H*_AC_ = 17 Oe, *f*_AC_ = 10 Hz) and a fixed electromagnetic microwave frequency of 2.10 GHz as a function of the thickness of polymeric slabs (*t*) overlapped from 0 to 6000 μm.

The addition of different polymeric slab thicknesses causes a variation in the effective permittivity (*ε*_eff_) [2,3,37,54], producing modifications in the *f*_min_ (Figure 6c). As the thickness of the layer increases, the *f*_min_ of the system decreases from ~2.37 GHz to ~2.07 GHz, being associated with an increase in the *ε*_eff_ of the medium. This is why ∆*S*_21_ vs. thickness (Figure 6c) has been determined tuning the fixed microwave frequency in the domain time measurement at the *f*_min_ of each thickness. When no polymer slab is used, the amplitude value of ∆*S*_21_ is close to ~−5.9 dB. In contrast, the ∆*S*_21_ increases considerably up to ~−24.2 dB, with an almost linear trend up to thickness (*t*) ~3100 µm. Beyond that, it reaches a stabilization level of ~−25.1 dB that could be considered as a broad system’s matching thickness [55,56]. The example presented shows the high versatility of the designed systems to obtain adjustable time-resolved variations up to ~−25.1 dB for the configuration 2. Surprisingly, the system has a high sensitivity to the progressive addition of polymer slabs with a thickness below 3100 µm (Figure 6c), indirectly obtaining the added thicknesses by knowing the AC applied magnetic field with a resolution of ~5.10^−3^ dB/µm. For sensor applications, it is important to widen the spectral effectiveness of the electromagnetic frequency near the antenna resonance exhibited. In this line, the time-resolved modulation is also investigated for frequencies that are located in the vicinity of the *f*_min_ (2.40 GHz). Figure 7a displays the effect of the polymeric slab thickness (*t*) on the ∆*S*_21_ modulation for five selected frequencies, varying from 2.00 GHz to 2.40 GHz (within the dipole scattering).

It can be observed that the ∆*S*_21_ difference shows a maximum for each frequency and it corresponds to the matching thickness under the experiment conditions. For 2.40 GHz, it is ~0 µm, coinciding with the minimum scattering (Figure 4b2). The rest of the chosen frequencies are as follows ~200 µm (2.30 GHz), ~1000 µm (2.20 GHz), and ~4000 µm (2.10 GHz). Concerning the fixed frequency of 2.00 GHz the matching thickness is located beyond 6500 µm. In the time-domain representation, the ∆*S*_21_ pulses are represented as an example at a fixed frequency of 2.10 GHz for six different thicknesses that ranged from 0 to 6000 µm (Figure 7b). This modulation exhibits a maximum amplitude of ~−18.1 dB for a thickness of 4000 µm, according to the results that are displayed in Figure 7a. Hence, the influence of remarkable magneto-impedance effects on the ∆*S*_21_ coefficient displayed by the configuration 2 is also confirmed for frequencies that are close to the *f*_min_ as a function of the thickness. Therefore, these kinds of measurements are, in fact, a way to experimentally obtain the matching thickness for a specific microwave absorber system at a fixed frequency located in the close environment of the dipole resonance. Accordingly, the resonant frequencies of a specific array configuration can be modified by fine-tuning the overlapping thickness.

### 3.4. Microwave Scattering Experiments Performed in Cu and Co-Based MWs Sensing Array Platforms by Applying DC Magnetic Fields

**Figure 8:**
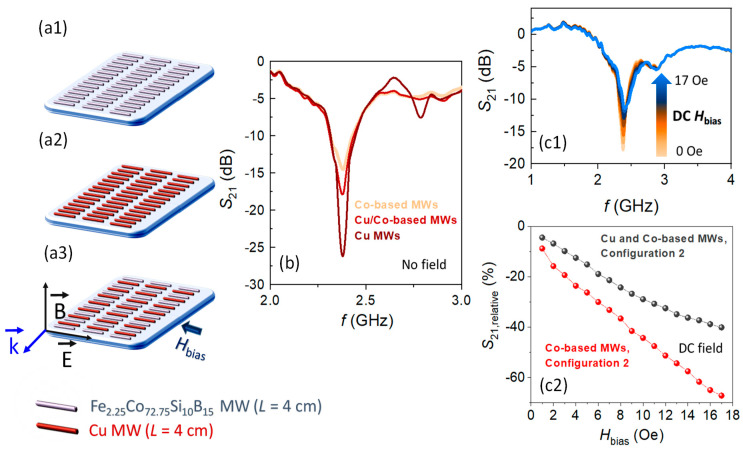
Influence of non-field and DC magnetic field conditions on the microwave scattering for the configuration 2: schematic illustration for sensing array platforms composed by (**a1**) Co-based MWs, (**a2**) Cu MWs, and (**a3**) a combination of Co-based and Cu intercalated MWs; (**b**) the *S*_21_ transmission coefficient obtained for the three cases under non-field conditions; (**c1**) the *S*_21_ transmission coefficient obtained under a DC magnetic field between 0 and 17 Oe for the combination of Cu and Co-based MWs arrays (combination (**a3**)), and (**c2**) *S*_21,relative_ value calculated for the combined system of Co-based and Cu MWs. The *S*_21, relative_ distribution of the combination (**a1**) with Co-based MWs is also added for comparison.

In an electromagnetic wave scattering that is produced by dipole antenna effects, it is well known that the energy that is scattered by the MW depends on its electrical conductivity [40,57]. As the electrical conductivity of the MW is higher, its scattered amplitude is larger, because the electric current is higher. In this sense, the present Co-based MWs show a value of *σ*_Co MW_ ~ 2.10^7^ S m^−1^, and it can be surpassed by some non-magnetic metallic elements, such as Cu. Therefore, it would be interesting to design combined Co-based and Cu MWs arrays to evaluate their response in the microwave electromagnetic scattering and, subsequently, to obtain their modulation through the application of DC or AC magnetic fields. For this purpose, Cu MWs with a diameter of ~100 µm are chosen, which correspond to a *σ*_Cu MW_ ~ 6.10^7^ S m^−1^ (Figure 8).

Configuration 2 is chosen as an example of potential technological application to exploit the proposed approach to improve the amplitude of microwave scattering maintaining the ability to be modulated with DC magnetic fields. For this end, array sensing platforms are fabricated to evaluate their microwave scattering properties while using Co-based MWs (Figure 8a1), Cu MWs (Figure 8a2), and a combination of alternating Co-based and Cu MWs (Figure 8a3). Under non-applied field conditions (Figure 8b), the resonant frequency *f*_min_ is located around ~2.38 GHz for the three sensing array platforms (*L* = 4 cm) confirming the antenna character of the Co-based MWs. In contrast, the resonance amplitude increases dramatically from ~−14.4 dB (Co-based MWs configuration) to ~−26.2 dB (Cu MWs configuration). For the array combination of alternating Cu and Co-based MWs, a minimum amplitude of the *S*_21_ transmission coefficient is recorded with an intermediate value between the previous configurations of ~−17.8 dB. However, the latter combination retains the advantage of being able to adjust the minimum amplitude through the application of relatively low strength magnetic fields due to the ultrasoft magnetic character of the Co-based MWs (Figure 8c1). In this line, Figure 8c2 shows the variation of the peak amplitude, when a DC magnetic field up to 17 Oe is progressively applied. For such a field, a minimum value of ~−11.2 dB is recorded. By calculating the values of *S*_21,relative_ (Equation (6)), a linear decreasing trend is also detected, as happened with the Co-based ones (combination (a1) in Figure 8), but with a lower slope reaching a maximum variation of ~40%. Such a reduction is expected, since only the Co-based MWs are affected by the DC magnetic field. However, these are interestingly high values of microwave attenuation keeping an amplitude of the recorded scattering significantly higher than the Co-based arrays. Furthermore, regardless of the DC-modulation character, this result might also be attractive for electromagnetic shielding applications, because this combination displays a microwave scattering of ~−11.2 dB at ~2.38 GHz, even with an external DC applied magnetic field with intensity of 17 Oe.

## 4. Summary and Conclusions

This work provides different MWs configurations to obtain a tunable and multiple response in the L and S bands (GHz). In addition, the suitability of different external stimuli, such as the application of AC and DC magnetic fields and the overlapping of variable thickness of polymer slabs, are demonstrated for the modulation of the microwave scattering coefficient. A ferromagnetic amorphous Co-based MW with low magnetostriction is the sensing material used, which presents high intensity electromagnetic scatterings that can be modulated by GMI effects. When MW linear arrays are fabricated, the amplitude of the microwave scattering is intensified and the resonant frequency *f*_min_ shifts towards lower frequencies as the MW length increases. Subsequently, three array configurations are designed and a substantial improvement in attenuation amplitude is achieved in configurations 2 and 3 with ~−16.8 and ~−47.9 dB, respectively. In addition, a large reduction of the FWHM of the band pass filter of ~0.03 GHz is obtained for configuration 3, narrowing the bandgap that is associated with the antenna resonance. When external DC and AC magnetic fields up to 17 Oe are axially applied through the MWs, the antenna resonance signature is progressively flattened and broadened. These effects are caused by the change in surface impedance produced when the MW switches from a circumferential anisotropy (maximum attenuation state) to an axial anisotropy (minimum attenuation state). With the DC field applied, variations of ~62%, ~67% and ~77% are obtained for configurations 1, 2, and 3, respectively. In contrast, an adjustable response in the time domain representation is also obtained when an AC magnetic field is applied. Furthermore, the addition of different thicknesses of overlapping polymeric slabs can be discerned with high sensitivity (5.10^−3^ dB/µm), due to the high resolution of the measurements. Interestingly, measurements as a function of overlapping polymeric thicknesses evidence the ability of these sensing array platforms to experimentally obtain different matching thicknesses at a fixed frequency within the dipole resonance range. These features open up the possibility of applying these configurations as overlapping thickness measurement platforms.

An advanced approach is also successfully tested by intercalating Cu MWs in the Co-based array to improve the intensity of microwave scattering, while maintaining the ability to tune its properties by the stimuli that are described above. As an application example, configuration 2 improves from ~−14.4 dB to ~−17.8 dB with Co-based MWs and with a Cu MWs intercalation in the Co-based one, respectively. In addition, a maximum difference of approximately 40% of the absorbed signal is obtained with an applied DC magnetic field with intensity of 17 Oe by maintaining an electromagnetic shielding of ~−11.2 dB at ~2.38 GHz.
